# Inter-observer agreement and diagnostic accuracy of myocardial perfusion reserve quantification by cardiovascular magnetic resonance at 3 Tesla in comparison to quantitative coronary angiography

**DOI:** 10.1186/1532-429X-15-25

**Published:** 2013-03-27

**Authors:** Katharina Ikuye, Dominik Buckert, Lisa Schaaf, Thomas Walcher, Wolfgang Rottbauer, Peter Bernhardt

**Affiliations:** 1 Department of Internal Medicine II, University of Ulm, Albert-Einstein-Allee 23, Ulm 89081, Germany

## Abstract

**Background:**

Quantification of cardiovascular magnetic resonance (CMR) myocardial perfusion reserve (MPR) at 1.5 Tesla has been shown to correlate to invasive evaluation of coronary artery disease (CAD) and to yield good inter-observer agreement. However, little is known about quantitative adenosine-perfusion CMR at 3 Tesla and no data about inter-observer agreement is available. Aim of our study was to evaluate inter-observer agreement and to assess the diagnostic accuracy in comparison to quantitative coronary angiography (QCA).

**Methods:**

Fifty-three patients referred for coronary x-ray angiography were previously examined in a 3 Tesla whole-body scanner. Adenosine and rest perfusion CMR were acquired for the quantification of MPR in all segments. Two blinded and independent readers analyzed all images. QCA was performed in case of coronary stenosis. QCA data was used to assess diagnostic accuracy of the MPR measurements.

**Results:**

Inter-observer agreement was high for all myocardial perfusion territories (ρ = 0.92 for LAD, ρ = 0.93 for CX and RCA perfused segments). Compared to QCA receiver-operating characteristics yielded an area under the curve of 0.78 and 0.73 for RCA, 0.66 and 0.69 for LAD, and 0.52 and 0.53 for LCX perfused territories.

**Conclusions:**

Inter-observer agreement of MPR quantification at 3 Tesla CMR is very high for all myocardial segments. Diagnostic accuracy in comparison to QCA yields good values for the RCA and LAD perfused territories, but moderate values for the posterior LCX perfused myocardial segments.

## Background

Visual assessment of perfusion cardiovascular magnetic resonance (CMR) at 1.5 Tesla has been shown to yield high diagnostic accuracy in comparison to coronary x-ray angiography for the detection of coronary artery disease (CAD) [1]. Its sensitivity and specificity is superior to single-photon emission computed tomography regarding the detection of CAD [2].

Perfusion CMR at 3 Tesla has increased signal-to-noise and contrast-to-noise ratios in comparison to 1.5 Tesla [3-6]. Moreover, maximum upslope for quantitative perfusion analysis has been proven to be increased at 3 Tesla [6]. These potential benefits at 3 Tesla have recently been shown to yield higher diagnostic accuracy in comparison to 1.5 Tesla for adenosine-perfusion CMR to detect CAD [7,8].

Intra- and interobserver agreement for the visual assessment of adenosine-perfusion CMR as well as interstudy reproducibility of quantitative assessment at 1.5 Tesla have been proven to be very high [9,10]. Moreover, quantitative analysis of adenosine-perfusion CMR at 3 Tesla exhibits a high correlation to invasively measured fractional flow reserve [11], which is regarded by many investigators as the standard diagnostic tool to evaluate hemodynamic significance of CAD. However, little is known about the inter-observer correlation and thus, reliability of the quantitative analysis approach of 3 Tesla perfusion imaging.

The aim of our study was to evaluate the inter-observer agreement of quantitative myocardial perfusion analysis at 3 Tesla and to assess its diagnostic accuracy in comparison to quantitative coronary angiography (QCA).

## Methods

### Study population

Sixty-three consecutive patients suspected for CAD or progression of known CAD, who were referred for diagnostic coronary angiography, were prospectively recruited. Patients were excluded, if they had a recent history of myocardial infarction (within 30 days), had previously undergone coronary artery bypass or prosthetic valve surgery, were medically unstable, and had contraindications for gadolinium-based contrast agents, adenosine infusion, or CMR. Study patients were asked to avoid caffeine or other methylxanthines for at least 24 hours before CMR. All patients underwent CMR within 72 hours before of coronary catheterization. The study was approved by the ethics committee of the institution. All participants gave written informed consent.

### CMR examination

All patients underwent CMR in a 3 Tesla whole-body system (Achieva, Philips Medical Systems, Best, Netherlands) using a 32-channel phased-array cardiac surface coil (Philips Medical Systems). Heart rate and blood pressure were monitored non-invasively during adenosine infusion. The CMR protocol used has been previously described in detail [8].

For functional analysis of the left and right ventricle a balanced steady-state free precession sequence was acquired in contiguous short-axis views covering the entire left and right ventricle from apex to basis (repetition time 3.4 ms, echo time 1.7 ms, acquired resolution 1.9 × 1.9 mm, flip angle α = 40°, slice thickness 8 mm, no interslice gap; acquisition in end-expirational breath-hold).

For perfusion imaging a spoiled gradient-echo sequence (repetition time 2.6 ms, echo time 1.3 ms, saturate pre-pulse with 100 ms delay, flip angle α = 18°, acquired resolution 2.5 × 2.5 mm, slice thickness 8 mm; acquisition in end-expirational breath-hold) was acquired in three short axis (apical, midventricular, and basal). After three minutes of adenosine infusion at a constant rate of 140 μg/kg/min, or earlier if angina pectoris was provoked, a bolus of 0.075 mmol/kg contrast agent (Dotarem, Guerbet, Villepinte, France) followed by 20 ml saline flush was administered with an injection rate of 5 ml/s. The sequence was repeated at rest ten minutes later using a second bolus of 0.075 mmol/kg contrast agent.

A 3D inversion-recovery gradient-echo sequence in short axis views for late gadolinium enhancement (LGE) visualization was acquired ten minutes after the second bolus of contrast agent (repetition time 7.1 ms, echo time 3.2 ms,, flip angle α = 15, acquired resolution 1.6 × 1.6 mm°, slice thickness 8 mm; navigator-based acquisition). The inversion time was individually adjusted for complete nulling of the myocardium.

### CMR analysis

Two experienced readers, blinded to patients’ data and angiographic results, analyzed the anonymized DICOM files. All images were analyzed on a separate workstation (ViewForum, Philips Medical Systems). Functional images were analyzed for end-diastolic and end-systolic volumes. Ventricular ejection fractions were calculated, respectively.

**Figure 1:**
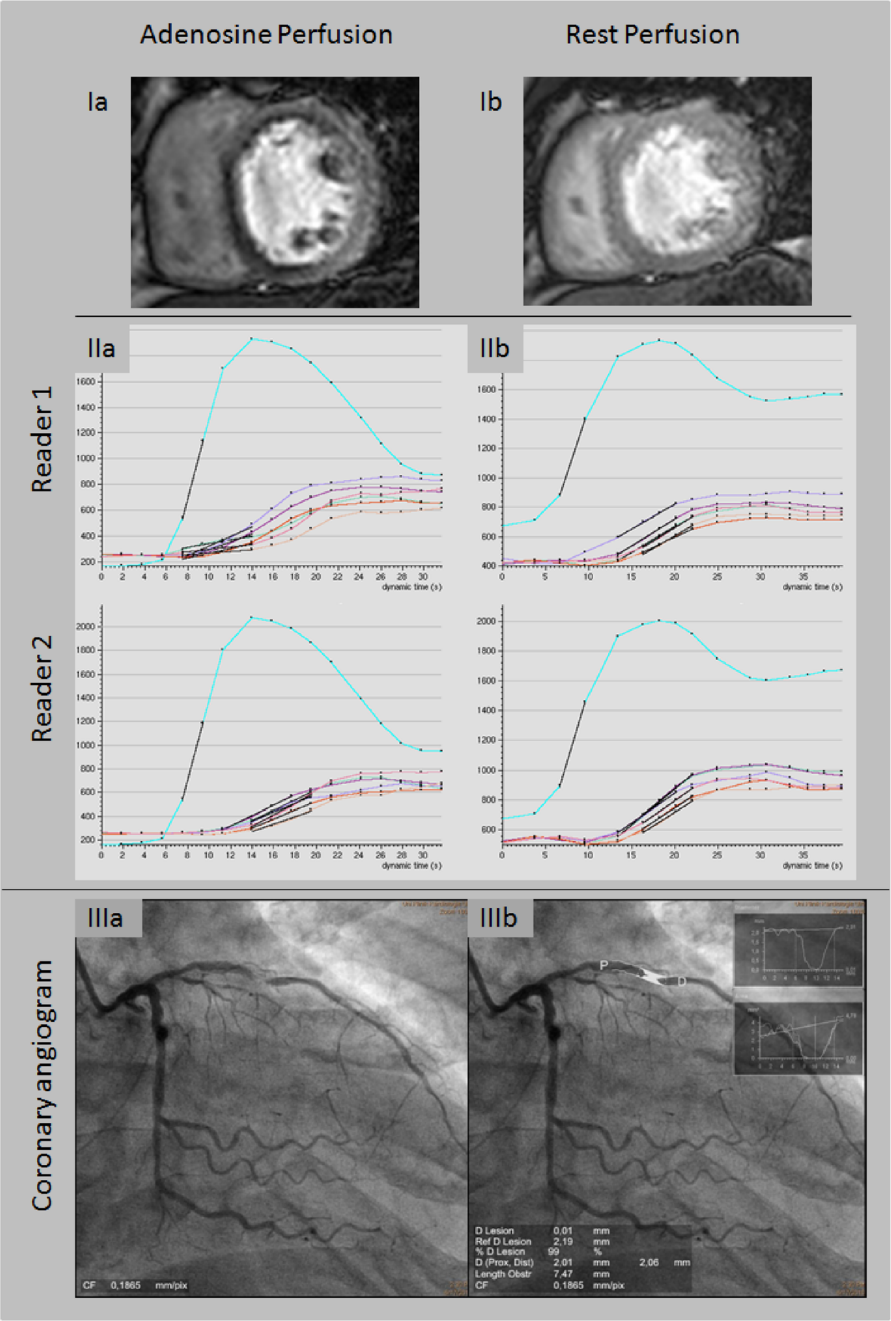
**Example of inducible ischemia during adenosine in segments supplied by the LAD (Ia) without corresponding perfusion deficit at rest (IIa).** Segmental upslope curves during adenosine and rest for both readers are shown in **IIa** and **IIb**. The coronary angiogram of the LAD stenosis and QCA of are provided in **IIIa** and **IIIb**, respectively.

For evaluation of myocardial perfusion reserve each reader drew the endo- and epicardial left ventricular contours manually in each adenosine and rest perfusion image after correction for motion using the same software (ViewForum, Philips Medical Systems) independently from the other reader. The myocardium was divided into 16 segments according to the recommendations of the American Heart Association [12]. The resulting signal intensity-time curves were adjusted for left ventricular signal intensity and baseline signal intensity as previously reported [11,13]. Myocardial perfusion reserve was than calculated dividing the segmental upslope during adenosine and rest [11,13]. Figure 1 provides an example of inducible ischemia during adenosine, corresponding segmental upslope curves during adenosine and rest for both readers, and respective angiogram including QCA of the LAD stenosis. The myocardial segments were assigned to the respective supplying coronary artery [14]. The mean myocardial perfusion reserve for all segment supplied by one coronary artery was calculated for further analysis. As previously validated against fractional flow reserve, we defined a myocardial perfusion reserve cut-off value of ≤1.3 as consistent with relevant myocardial ischemia [11].

### Quantitative coronary angiography

Coronary angiography was performed within 48 hours after CMR in accordance to the ACC/AHA guidelines [15]. In case of coronary artery stenosis in a coronary artery with a diameter ≥2 mm quantitative analysis was performed by an experienced reader blinded to patients’ data, clinical symptoms, and CMR results using commercially available standard software (CAAS 5.9, Pie Medical Imaging, Maastricht, Netherlands). A threshold of ≥70% luminal narrowing was used to identify significant coronary artery stenosis [16].

### Statistical analysis

Continuous variables were tested by the two-tailed *t* test after being tested for normal distribution by the D’Agostino-Pearson test. They are reported as mean value ± standard deviation. Categorical data is presented as number (%) and compared using the Fisher’s exact test.

Diagnostic accuracies of both readers in comparison to the quantitative coronary angiographic results were tested using receiver-operating characteristics curve analyses.

Inter-observer agreement was being tested using Spearman’s coefficient of correlation (ρ). Additionally, the correlation coefficient r was calculated. A p value <0.05 was considered significant for all tests.

## Results

### Study population

Six patients had to be excluded due to obesity (N = 3), previous unknown coronary artery bypass surgery (N = 1) and uncompleted CMR exam due to technical issues (N = 2). Thus, our study population consisted of 53 patients. Mean age was 63.0 ± 9.3 years; 68% of our patients were male. Patients’ characteristics including cardiovascular risk factors, Framingham 10 years risk for cardiovascular events, history of CAD, and medication are provided in Table 1.

**Table 1:** Study population

	**N = 53**
Age, years	63.0 ± 9.3
Female gender, female (%)	17 (32.1)
Body mass index, kg/m^2^	27.4 ± 3.5
**Cardiovascular risk factors**	
Hypertension, N (%)	42 (79.2)
Hypercholesterolemia, N (%)	36 (67.9)
Diabetes, N (%)	11 (20.8)
Smoking, N (%)	8 (15.1)
Framingham 10 years risk,%	13 ± 7
**History of coronary artery disease**	
Known coronary artery disease, N (%)	34 (64.2)
Known myocardial infarction, N (%)	18 (34.0)
**Medication**	
β- Blocker, N (%)	34 (64.2)
AT1-Inhibitor/ACE-Inhibitor, N (%)	39 (73.6)
Statin, N (%)	40 (75.5)
Platelet aggregation inhibitor, N (%)	46 (86.8)
**Ventricular volumes and function**	
Left ventricular ejection fraction,%	61 ± 9
Left ventricular end-diastolic volume index, ml/m^2^	81 ± 15
Left ventricular mass index, g/m^2^	54 ± 10
Right ventricular ejection fraction,%	63 ± 6
Right ventricular end-diastolic volume index, ml/m^2^	72 ± 14
**Quantitative coronary angiography**	
Patients with stenosis ≥ 70%, N (%)	25 (47.2)
1-vessel disease	15 (28.3)
Multi-vessel disease	10 (18.9)

### CMR

No major complications were observed during CMR. The results of the left and right ventricular volumetric analysis are provided in Table 1. During adenosine a significant decrease of systolic and diastolic blood pressure could be observed in our patient cohort (Table 2). Furthermore, heart rate and rate pressure product increased, significantly.

**Table 2:** Hemodynamics

	**Rest**	**Adenosine**	**p**
Systolic blood pressure (mmHg)	133 ± 20	127 ± 20	.0063*
Diastolic blood pressure (mmHg)	76 ± 14	72 ± 14	.0434*
Heart rate (beats per minute)	64 ± 10	80 ± 13	<.0001*
Rate pressure product	8,480 ± 1,710	10,180 ± 2,304	<.0001*

* statistically significant.

**Figure 2:**
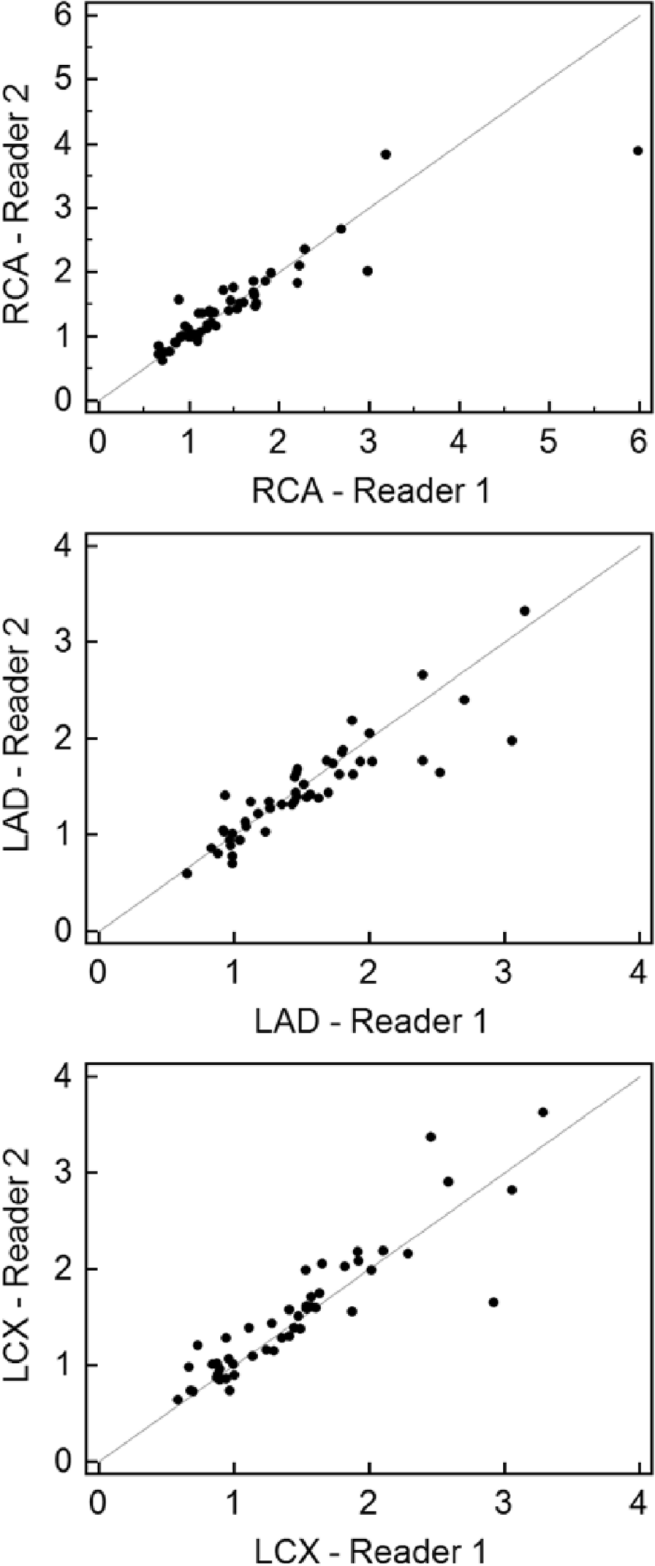
Scattergrams of myocardial perfusion reserve indices the RCA with a correlation coefficient of r = 0.91 (p < 0.0001), the LAD with r = 0.91 (p < 0.0001), and the LCX with r = 0.90 (p < 0.0001) territories.

In four patients the image quality was insufficient for quantitative perfusion analysis. Latter mentioned patients were excluded from further analysis. In two patients a total of four segments had to be excluded from quantitative perfusion due to interference of the left ventricular outflow tract. Mean myocardial perfusion reserve indices of both readers per perfusion territory are provided in Table 3. Spearman’s correlation coefficient yielded ρ = 0.92 for the RCA and LAD perfused territories and 0.93 for the territories supplied by the LCX. All Spearman’s correlation coefficients achieved statistical significance. Figure 2 shows scattergrams of myocardial perfusion reserve indices in all perfusion territories with calculated correlation coefficient of r = 0.91 (p < 0.0001) in the RCA, r = 0.91 (p < 0.0001) in the LAD, and r = 0.90 (p < 0.0001) in the LCX perfused territories.

**Table 3:** MPR values for each reader and perfusion territory including Spearman’s correlations coefficient

**Perfusion territory**	**MPRi reader 1**	**MPRi reader 2**	**Spearman’s correlation coefficient (ρ)**	**p**
RCA	1.47 ± 0.66	1.49 ± 0.87	0.92 (95% CI 0.86-0.95)	<.0001*
LAD	1.47 ± 0.51	1.53 ± 0.57	0.92 (95% CI 0.85-0.95)	<.0001*
LCX	1.52 ± 0.66	1.45 ± 0.64	0.93 (95% CI 0.88-0.96)	<.0001*

*MPRi* myocardial perfusion reserve index.

*RCA* right coronary artery.

*LAD* left anterior descending.

*LCX* left circumflex.

* statistically significant.

### QCA and CMR diagnostic accuracy

Coronary angiography was performed in all patients without major complications. QCA revealed a coronary stenosis ≥70% in 25 (47.2%) patients (see Table 1). The RCA was affected in 13, the LAD in 11, and the LCX in 11 patients, resulting in 15 one-vessel and 10 multi-vessel diseases.

**Figure 3:**
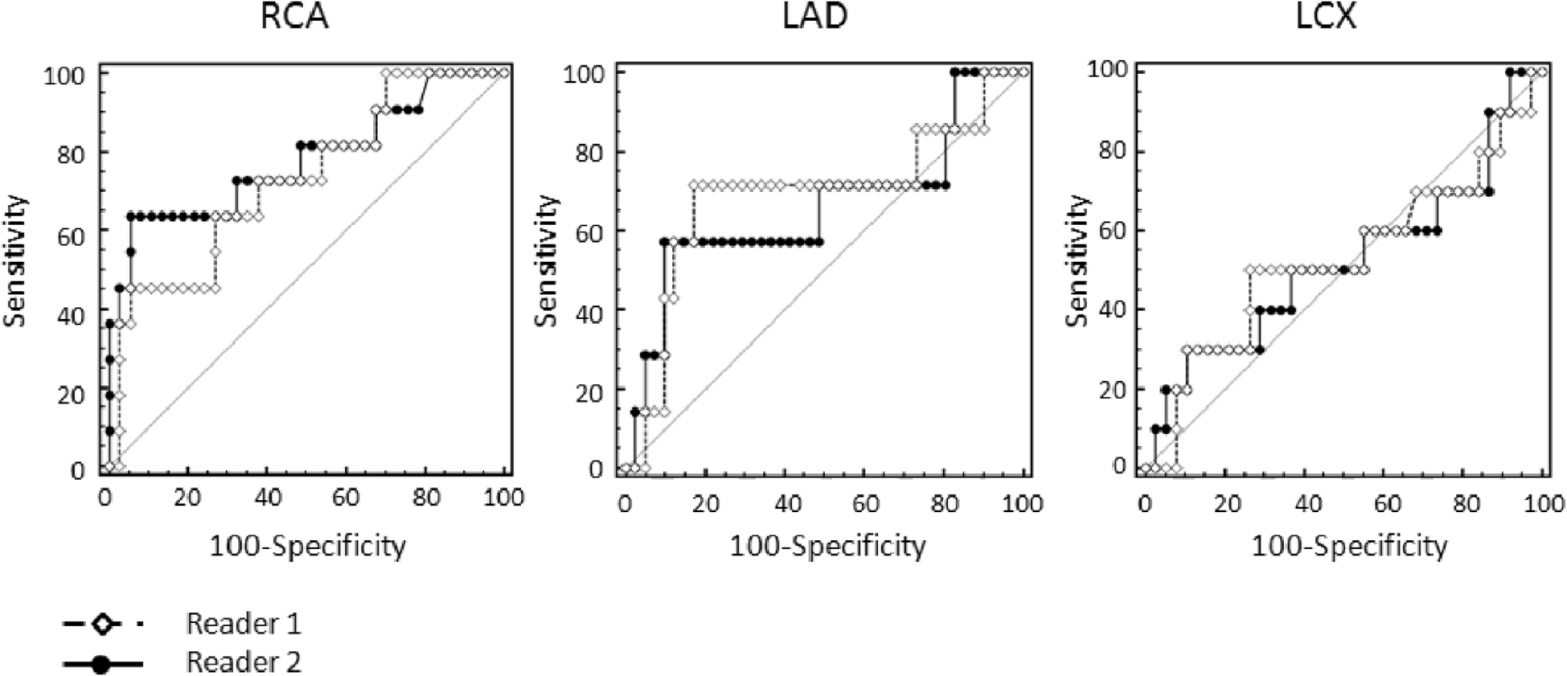
**Receiver operator characteristic (ROC) analysis of myocardial perfusion reserve quantification of both readers with an area under the curve of 0.78 and 0.73 for RCA, 0.66 and 0.69 for LAD, and 0.52 and 0.53 for LCX perfused territories, respectively.** There were no significant differences between both readers.

Receiver operator characteristic (ROC) analysis of myocardial perfusion reserve quantification of both readers yielded an area under the curve of 0.78 and 0.73 for RCA, 0.66 and 0.69 for LAD, and 0.52 and 0.53 for LCX perfused territories, respectively. There were no significant differences between both readers. ROC curves for all perfusion territories are shown in Figure 3. Sensitivity, specificity, and overall accuracy for both readers are provided in Table 4.

**Table 4:** Diagnostic accuracy of both readers in comparison to quantitative coronary angiography

	**Sensitivity**	**Specificity**	**Overall accuracy**
**Reader 1**			
RCA	.82	.46	.62
LAD	.71	.44	.56
LCX	.50	.45	.48
**Reader 2**			
RCA	.82	.41	.60
LAD	.71	.46	.57
LCX	.60	.42	.51

## Discussion

There is little data about inter-observer agreement of quantitative perfusion assessment at 1.5 Tesla, and this is to the best of our knowledge, the first study to report inter-observer agreement of quantitative myocardial perfusion analysis at 3 Tesla. We were able to demonstrate a high inter-observer agreement of quantitative myocardial perfusion reserve performed at 3 Tesla. The available studies at 1.5 Tesla report an inter-observer agreement of kappa = 0.66 [17]. Other studies yielded inter-observer agreements of 0.73 [9] and r = 0.93 [18]. Due to the large number of artifacts that can occur in 3 Tesla CMR [19], the question arises, whether inter-observer reproducibility of quantitative perfusion analysis could be equal to that observed at 1.5 Tesla. Our data prove a high inter-observer agreement for all coronary perfusion territories.

The diagnostic accuracy observed in our study showed good values for RCA, reduced accuracy for LAD, and poorer accuracy for LCX perfused myocardial territories. This is in concordance to similar observations with best values for RCA and moderate values for LCX supplied segments [20]. Latter study, however, evaluated qualitative myocardial perfusion assessment in comparison to QCA. The observation of reduced diagnostic accuracy in the posterior regions is probably caused by a poorer signal-to-noise ratio in those segments due to the distance to the surface coil [9,21].

A recent study proved a poor correlation of r = 0.58 between QCA and fractional flow reserve [20]. However, quantitative analysis of myocardial perfusion reserve at 1.5 [21] and 3 Tesla [11] has been shown to yield very high diagnostic accuracy in comparison to fractional flow reserve which is regarded by many investigators to be a very sensitive diagnostic tool to measure functional significance of coronary artery stenosis, whereas QCA does only provide anatomical, but no functional information about the stenosis severity. This opinion is reasonable, since it has been lately shown that fractional flow reserve is superior to QCA driven coronary intervention in preventing myocardial infarction, revascularization or death [22,23]. Myocardial perfusion reserve is similar to fractional flow reserve a diagnostic tool to measure the functional significance of CAD. Hence, the poor correlation to QCA is understandable. Quantitative CMR myocardial perfusion assessment could thus serve as a non-invasive surrogate to fractional flow reserve to measure the functional significance of CAD, as it already has been shown to yield high diagnostic accuracy in comparison to fractional flow reserve [11].

### Limitations

Patients who previously had undergone coronary artery bypass or prosthetic valve surgery were excluded from the study. Hence, this might be a limitation because the results of the study at hand might not be translatable to this population of patients.

Moreover, we had to exclude four patients from quantitative perfusion analysis because of poor image quality. In two other patients a total of four segments were excluded due to interference of the left ventricular outflow tract. This was done to allow for good and reliable quantitative perfusion analysis. However, this is another possible limitation to our study in terms of selection bias.

## Conclusions

Quantification of myocardial perfusion reserve at 3 Tesla yields very high inter-observer agreement, as could be shown in the present study. Diagnostic accuracy in comparison to quantitative coronary analysis for the LAD and RCA perfused myocardial territories is good and moderate for the LCX perfused territories.

## Abbreviations

CMR: Cardiovascular magnetic resonance; CAD: Coronary artery disease; QCA: Quantitative coronary angiography; LGE: Late gadolinium enhancement.

## Competing interests

The authors declare that they have no competing interests.

## Authors’ contributions

KI analysis and interpretation of data, drafting the manuscript. TW analysis and interpretation of data. LS analysis and interpretation of data. DB analysis and interpretation of data, drafting the manuscript. WR final approval of the manuscript, revising the manuscript critically for important intellectual content. PB conception and design, analysis and interpretation of data, drafting the manuscript. All authors read and approved the final manuscript.
